# Lessons Learned from Exploring Sexual Health Among Migrant and Refugee Women and Men in South Australia

**DOI:** 10.3390/healthcare14081065

**Published:** 2026-04-17

**Authors:** Negin Mirzaei Damabi, Patience Castleton, Bridgit McAteer, Zohra S. Lassi

**Affiliations:** 1 Robinson Research Institute, Adelaide University, Adelaide 5000, Australia; negin.mirzaeidamabi@adelaide.edu.au (N.M.D.); patience.castleton@adelaide.edu.au (P.C.); 2 School of Public Health, College of Health, Adelaide University, Adelaide 5000, Australia; 3 Survivors of Torture and Trauma Assistance and Rehabilitation Service, Adelaide 5000, Australia

**Keywords:** migrant health, sexual and reproductive health, healthcare experience, reflection, women’s health, men’s health

## Abstract

**Background**: Sexual health research with migrant and refugee communities presents unique challenges, shaped by cultural sensitivities, stigma, and the under-representation of these populations in health research. However, lived experiences insights are essential for the development of appropriate and useful research and health initiatives. It is important to learn from researchers’ experiences to expand the representation of migrant and refugee community voices. **Method**: This paper draws on two qualitative studies conducted in South Australia: one exploring the sexual and reproductive health perspectives of refugee and migrant women, and the other of men. We reflect upon the methodological and ethical considerations in conducting research in this sensitive field and provide recommendations for future researchers and healthcare providers when working with migrant and refugee communities. **Results**: Both studies encountered difficulties in relation to participant recruitment, cross-cultural communication, and addressing taboos surrounding sexual health. At the same time, they highlighted opportunities for generating meaningful insights through culturally safe, gender-sensitive approaches and collaboration with community stakeholders. **Conclusions**: By synthesising experiences from both projects, we identify practical strategies for building trust, overcoming linguistic and cultural barriers, and creating supportive environments for discussing sensitive topics. These reflections offer guidance for researchers and clinicians aiming to advance culturally responsive sexual health research and strengthen healthcare provision for migrant and refugee populations.

## 1. Introduction

More than 30% of Australia’s population is from migrant backgrounds, with over 91,000 identifying as asylum seekers by the end of 2024 [1]. Humanitarian migrants in Australia experience a range of intersecting structural and individual challenges when accessing appropriate healthcare services [2]. Limited English proficiency, financial pressures, low health literacy, cultural beliefs, competing settlement priorities and discrimination within healthcare settings all impede regular and positive engagement with services [3]. These challenges contribute to the reduced utilisation of primary and preventive healthcare and, in turn, the subsequent high burden of Sexual and Reproductive Health (SRH) concerns [4,5,6].

Research indicates low SRH awareness and education amongst many migrants [7,8,9], with many finding it difficult to source helpful information about SRH topics, including contraception and service availability [5,10]. These difficulties are commonly attributed to difficulties in sourcing information in appropriate languages and cultural contexts, low health literacy skills and shame and taboo encircling SRH topics [11,12].

In addition to the structural challenges faced in accessing healthcare, strict cultural and patriarchal values and beliefs often prescribe gender roles for men and women that further limit their understanding of SRH and their abilities to access services post migration [7]. Cultural and religious norms that suppress open discussion of SRH can prevent many culturally diverse men and women from seeking SRH education or healthcare support. Men and women are similarly impacted by gender norms and SRH taboo, with studies showing that refugee men commonly see SRH as un-masculine and exclusively female problems [5]. These often deep-rooted beliefs carried post migration impede their knowledge of SRH concerns and limit their confidence to seek medical assistance. However, research that amplifies the voices of men from migrant backgrounds is limited, thus impeding the depth of our knowledge and understanding of the impacts of these beliefs on their SRH.

Moreover, research on migrant health is inherently constrained by the self-selected nature of migrant and refugee populations. As noted in previous literature, migrants and refugees are not altogether representative of their populations of origin, often being healthier and having more education at the time of migration [13]. Thus, the health status, behaviours and beliefs of migrant and refugee popilations may reflect pre or post migration characteristics rather than the conditions in the host country. Qualitative research detailing these potential differences is also essential to gain a deeper understanding of such dynamics and to contextualise disparities in health behaviours.

Additionally, the literature indicates that an estimated 44% of asylum-seeking women worldwide have experienced sexual violence pre or post migration [14], further contributing to the sensitive nature of SRH topics amongst refugee populations. Research has also indicated lower levels of sexual desire and arousal amongst migrant women compared to their native counterparts. This further suggets that migration experiences may suppress sexual enjoyment [6]. However, research in this space is extremely limited, with the lived experiences of migrant and refugee women often overlooked or unheard of in existing literature.

Despite the lack of existing research, the literature has consistently shown the importance of lived experiences insights when recommending and implementing positive and useful change in healthcare and policy settings [15,16]. Therefore, it is vital to continue detailing refugee experiences within healthcare systems in Australia, particularly within SRH spaces, through qualitative research approaches. However, as previously discussed, individuals from refugee backgrounds often face pre and post migration disadvantages that leave them in uniquely vulnerable positions of threatened autonomy [17]. Therefore, whilst involving migrants and refugees in research is essential to informing targeted services and programs, carefully designed ethical and methodological approaches are required when inviting and involving refugee participants.

It is particularly important for researchers to learn from their own experiences, and from others’ experiences, when discussing SRH with refugee men and women in research settings. However, researchers rarely examine how their methodological choices, recruitment strategies, language approaches, interview formats, and disclosure timing shape what participants feel able to share. Maintaining reflexivity throughout the research process is therefore critical to ensuring that ethical, culturally safe, and supportive environments are created for participants.

This paper, therefore, examines the relationship between research methodology and knowledge production in sexual health research with migrant and refugee populations. We draw on two qualitative studies conducted in South Australia: one exploring sexual function among 20 migrant and refugee women [18], the other examining SRH care among 21 migrant and refugee men (unpublished). Rather than simply reporting what participants shared, we analyse how our methodological approaches shaped what could be learned. Specifically, we examine how recruitment strategies determined who participated, how language choices influenced disclosure, how interview formats affected what could be articulated, and what remained unspeakable despite our efforts to create supportive research environments. In doing so, we aim to inform future researchers and academics of the barriers and facilitators encountered when proactively and meaningfully engaging refugee and migrant men and women in SRH research.

## 2. Methodology

This reflective paper draws on two parallel qualitative studies exploring sexual health among migrant and refugee populations in South Australia. Semi-structured qualitative interviews were selected as the data collection method for both components. This structure allowed participants to share experiences in their own words whilst enabling the researcher to follow unexpected but important lines of inquiry, an approach particularly suited to sensitive topics where participant-led disclosure is essential [19]. All participants provided informed consent for their interview data to be used in primary and secondary research associated with the original studies. The women’s component [18] examined sexual function experiences, whilst the men’s component, currently undergoing final revisions for publication, explored sexual and reproductive health within the Australian healthcare system.

### 2.1. Women’s Component

The women’s component recruited 20 reproductive-aged women (18–49 years) who identify as cisgender and heterosexual, had migrated from low- and middle-income countries, and were sexually active (defined as having had sexual intercourse within the past four weeks) [18]. This focused eligibility reflected the study’s aim to explore sexual function changes before and after migration. A sample of 20 participants was determined appropriate for a qualitative study of this scope, consistent with thematic saturation principles in reflexive thematic analysis, where depth of engagement is prioritised over sample size [20]. Recruitment employed multiple pathways: flyers distributed through six community organisations (Australian Migrant Resource Centre, International Organisation for Migration, Refugee Advocacy Service, Australian Refugee Association, Vinnies Refugee & Asylum Seeker Service, FBW Gynaecology Plus Clinic), social media outreach through institutional networks, and a purpose—the Refugee and Immigrants Sexual Health Endeavour (RISE) website (https://neginmirzaeidamab.wixsite.com/rise-2 (accessed on 8 March 2024)) featuring multilingual information and online booking. All recruitment materials were explicitly transparent about the sexual health focus from the outset.

Semi-structured interviews lasting 40–60 min were conducted in person or virtually. The interview guide, informed by a prior scoping review and Berry’s Acculturation Theory, consists of demographic questions followed by a thematic exploration of sexual function experiences. The lead researcher, who has a migrant background, multilingual capabilities (fluent in Persian/Farsi, comfortable in Urdu, Hindi, Dari), and clinical experience as a midwife, conducted all interviews. Professional interpreters were available for other languages.

The team explicitly acknowledged the ‘double-edged sword’ of shared migrant identity: whilst it facilitated trust, it risked assumptions about shared experiences. To address this, the study emphasised open-ended questioning, maintained reflexive journals, and explicitly valued each woman’s unique perspective.

Data were analysed using reflexive, inductive thematic analysis following Braun and Clarke’s six-phase framework, with NVivo facilitating data management. Reflexive thematic analysis was selected over other analytical approaches because it is epistemologically consistent with a critical reflexivity framework, explicitly acknowledging the researcher’s active role in knowledge production rather than treating analysis as a neutral process.

### 2.2. Men’s Component

The men’s component recruited 21 men through social media and flyers placed in refugee community organisations and visa help centres, with strong support from Survivors of Torture and Trauma Rehabilitation Services (STTARS). This population was selected to address a significant gap in the literature: whilst migrant women’s sexual and reproductive health has received some research attention, the experiences of migrant and refugee men remain largely absent from the evidence base.

The men’s component was focused on all aspects of healthcare access and understanding of health post migration to Australia. Therefore, initial recruitment material looked different than that in the women’s component, with flyers using plain text and a large font to invite men to discuss their experience with all healthcare services in Australia. A consultation with STTARS nurses assisted in determining how to sensitively approach SRH topics upon initial contact with prospective participants. The research team explicitly informed the men that interviews would cover topics of all healthcare services, including SRH and mental health, providing them with the participant information sheet that provided further details regarding the interview structure. Prospective participants were asked to confirm their interest in participation before consent forms were sent and interviews were scheduled. No men decided against participation.

Eighteen of 21 men requested online interviews, with many initially keeping cameras off. To prevent duplicate participation whilst respecting anonymity, cameras were required only for introductions, then participants could turn them off. None requested male interviewers, and only two Spanish-speaking participants requested translators.

Following STTARS recommendations, interviews adopted a gradual approach, starting with broad healthcare questions before slowly integrating sexual and reproductive health topics. STTARS also recommended a casual format to minimise anxiety. The friendly atmosphere allowed participants and interviewers to joke and laugh together, building trust and easing pressure around sensitive discussions.

Researchers reflected on each interview through transcript review, evaluating vocabulary sensitivity and noting improvements for subsequent interviews. Support phrases like ‘tell me more’, ‘that’s really interesting’, and ‘thank you for sharing’ were naturally used throughout, with consistently positive participant responses.

### 2.3. Reflexive Practice

Reflexivity is a key component of qualitative work, ensuring researchers are aware of, and continually critically examining, the influence that their own background, assumptions and personal beliefs have on data interpretation [21]. Reflexive analysis was conducted independently for each study prior to comparative analysis for this reflective piece based on the framework described by [21]. The two components emphasised different reflexivity dimensions due to the differing personal and research backgrounds of the interviewers. The women’s study focused on researcher positionality, explicitly addressing the ‘double-edged sword’ of shared migrant identity through constant awareness and reflexive journaling. The lead researcher for the women’s component, a female migrant, brought shared cultural and gendered understandings of the research topic that positively contributed to discussions between the participant and interviewer, eliciting comfort and trust.

In contrast, the men’s study focused on interview technique, evaluating vocabulary sensitivity and identifying effective supportive phrases through transcript review as interviews were undertaken by a young Australian female (PC). Ongoing consultation with men from culturally diverse backgrounds and members of the partner organisation STTARS were made to ensure culturally informed practices were maintained.

Both approaches offered value, addressing different aspects of conducting ethical qualitative research. Although conducted separately for the male and female components, researchers across both teams engaged in ongoing reflective discussions. These collaborative consultations ensured that individual assumptions, positionalities and lived experiences were continually examined and critically interrogated in relation to the interpretation of the findings.

Reflexive analysis was an imperative tool used during the collaborative analysis for this reflexive piece. Lead researchers NMD and PC comparatively reflected on their unique experiences in the two studies, identifying similarities and differences in their role as researchers and interviewers, interactions with participants and interpretive findings. It was important for the researchers to ensure that differences in gender dynamics were not in fact artefacts of the differing methodological designs, thus NMD and PC engaged in extensive collaborative reflection on their interview techniques. The different cultural backgrounds and research experiences and expertise of NMD and PC provided disciplinary perspectives to data interpretation. However, through collaborative analysis of how individual perspectives shaped data collection, coding and interpretation of the original studies, this reflexive piece synthesises these insights to highlight how our positionalities influenced the research process. Reflexive lenses to interview processes and findings are evident throughout the following results section.

## 3. Results

### 3.1. Recruitment Outcomes: Different Approaches, Similar Success

The women’s component successfully recruited 20 participants from countries including Pakistan, India, China, Nigeria, Cameroon, Zimbabwe, Afghanistan, and Iran. Length of stay in Australia ranged from 1 to 11 years with a median of 5 years. Visa statuses varied: three citizens, seven permanent residents, nine temporary residents, and one refugee.

The men’s component recruited 21 participants, similarly representing diverse migration backgrounds and experiences: Colombia (1), Congo (2), Ethiopia (1), Gambia (2), Ghana (2), Iran (1), Iraq (1), Kenya (1), Myanmar (1), Nepal (1), Nigeria (1), Pakistan (2), Sudan (2), Syria (1) and Venezuela (1). Length of stay in Australia was 1–10 years with a median of 6 years. Visa statuses were mainly humanitarian and refugee (12), with others on family reunion (4), temporary protection (4), and prospective marriage visas (1).

The comparable recruitment success (20 women, 21 men) despite markedly different disclosure strategies suggests that neither upfront transparency nor delayed disclosure of the inclusion of SRH topics is inherently superior. Rather, the optimal approach appears to depend on specific cultural contexts, gender considerations, and community partner expertise.

### 3.2. Partnership Dynamics

Whilst the women’s component partnered with six organisations for broad community reach, the men’s component developed an intensive relationship with STTARS that extended beyond recruitment to methodological consultation. STTARS nurses advised omitting sexual health from initial promotional materials, offering camera-off options, adopting casual interview tones, and gradually approaching sensitive topics. This guidance proved invaluable, and STTARS’ endorsement carried substantial weight with men who viewed researchers as connected to an organisation that had previously supported them. Whilst the partnership dynamics were different, both methods achieved similar goals of recruitment through trusted networks and provision of resources and advice on research methods.

The RISE website in the women’s component serves as a digital innovation allowing private, multilingual access to study information. However, it requires substantial resources and may have excluded women with limited digital access.

### 3.3. The Language Paradox

Despite extensive multilingual support in both components, nearly all participants chose English. All 20 women chose English despite team fluency in Urdu, Persian, Hindi, and Dari, and professional interpreters available for other languages. Only two of 21 men requested translators.

Women later explained this choice. Some described English as creating emotional distance: ‘In English, it’s like I’m a different person, more professional, more open.’ Another said discussing sexual topics ‘feels wrong’ in her native language but more acceptable in English. English appeared to create psychological space between their ‘traditional self’ and ‘Australian self’. As a Persian-speaking migrant woman herself, NMD was struck by how familiar this feeling was; she had navigated the same linguistic split in her own life. This recognition deepened her understanding of what participants were describing, whilst also prompting her to remain cautious about assuming her experience mirrored theirs.

Contrastingly, the men described their want to speak English as an opportunity for them to practice their skills or simply felt that their English skills were developed enough to participate in the research in English.

Yet men and women simultaneously identified language as a healthcare barrier, with many men mentioning their preference to see healthcare providers who can speak their native language: ‘Language is the first barrier.’ This paradox reveals the complex relationship between language preference, identity, and context.

### 3.4. Anonymity and Interview Format

Eighteen of 21 men requested online interviews, with many initially keeping cameras off. This camera-off option appears to provide psychological safety through visual anonymity. The women’s component also offered flexible formats, though uptake was not quantified.

Interestingly, no men requested male interviewers despite being offered this option. Some male participants further went on to note that they did not have a gender preference in healthcare, rather seeing all healthcare workers as professionals who are trained the same. The women’s component did not offer interviewer gender choice, though women later expressed strong preferences for female healthcare providers.

### 3.5. Interview Atmosphere

The two components established different atmospheres during the interviews. The men’s study explicitly adopted a casual format with jokes and laughter, building trusting relationships that eased pressure. Men were described as ‘enthusiastic’ participants responding positively to reassuring phrases from the interview. Despite many interviews being conducted on zoom, the men were audibly engaged and willing to discuss many experiences and aspects of their health and migration journeys. PC reflected that this casualness was partly strategic and partly instinctive, without shared cultural or migration experience, building warmth through humour felt like the most honest way to close the distance between herself and participants.

The women’s study emphasised gentle support, normalising discomfort, respecting boundaries, and patience with silence. Women’s responses suggested more hesitation and emotional complexity, with some becoming tearful or providing brief responses without elaboration. NMD reflected that she sometimes found silence more difficult to sit with when she sensed a woman was holding back something she herself might have struggled to express. This awareness prompted her to be especially careful not to fill silences too quickly or steer participants toward disclosures they were not ready to make.

Whether these differences reflected actual variations in interview tone, gender dynamics, or simply different documentation emphases remains unclear. It was important to ensure that any differences in gender dynamics were not simply artefacts of the two studies’ different designs. N.M.D. and P.C. therefore spent considerable time reflecting together on their interviewing techniques before drawing any comparative conclusions. 

### 3.6. Topics That Remained Difficult

Certain topics proved extremely challenging in the women’s component. Many women described ‘living double lives’, presenting as modest to family whilst having relationships families were unaware of. One woman reflected: ‘Being 35 and having my first relationship here, I feel like a teenager sometimes. But I also carry all these years of cultural programming. I often feel guilty, even though I know I’m not doing anything wrong.’ NMD noted in her reflexive journal that this account resonated with her personally, and that managing this resonance, staying present with the participant without projecting her own feelings onto the narrative, required conscious effort during and after the interview.

Female Genital Mutilation presented particular challenges, with many expressing discomfort discussing it. None differentiated between WHO classifications. Discussions remained brief and general rather than personal.

Orgasm emerged as challenging not from taboo but from fundamental confusion. One woman stated: ‘I didn’t understand what orgasm actually is. So, I had to research it.’ Another explained: ‘I know I can definitely get an orgasm by myself. I can also get good pleasure from sex with my partner, but it feels different… I’m not entirely sure if that means I don’t get an orgasm from sex, or it’s just that there are different types of orgasm.’ This confusion reflected limited sexual education and absence of intergenerational knowledge transfer.

The men’s component noted a different experience. No specific topics were asked of the men, however many seemed to find difficulties in initially opening up to the interviewer about SRH experiences. As previously described, interviews began with discussions about general healthcare with a slow transition to sensitive topics of SRH. After first prompting mention of SRH, most men were open and receptive to continue discussing SRH through each question, however some men shied away from the topic, only discussing it if prompted by the interviewer. Some men opened up about personal struggles with sexually transmitted infections and experiences visiting sexual health clinics, comfortably engaging in discussions around their personal SRH experiences. Additionally, whilst all choosing to undertake the interview in English, many men found difficulties in articulating their SRH experiences in English, many pausing repetitively to ‘find the English word’.

### 3.7. Communication Beyond the Interview

Women described significant difficulty communicating sexual needs with partners, often using non-verbal strategies. One explained: ‘If we’re in bed and something he did actually hurts me, I’ll stop him, but I’m not going to say much about it. I’ll just pull away and hope that over time, he’s going to learn not to do it in a certain way.’

Fear of causing partner distress prevented discussions of dissatisfaction: ‘I just have the fear of the unknown because if I say, I don’t experience orgasm, maybe they could blame themselves.’

NMD reflected that hearing women describe these silences and recognising versions of them from her own cultural context reinforced for her how much this research was documenting not individual dysfunction but the embodied consequences of structural and cultural silencing. That recognition informed how she coded and interpreted these accounts in the original study, and shapes the framing of this reflective paper.

This was not a topic discussed in the male component.

### 3.8. Healthcare Barriers

Women reported never discussing sexual concerns with providers due to shame (‘I was too shy to reach out to doctors. I just tried to search online’), financial constraints (‘They are expensive as a migrant who doesn’t have Medicare. Sometimes I have more urgent needs rather than my orgasm problems’), lack of awareness (‘I’ve never heard of that [sexual health clinic]’), and systemic barriers (‘Some GPs and specialists are not taking new patients’).

Similar barriers were shared with the male participants, with most also reporting financial constraints (‘There are many costs involved, especially with specialists’), lack of awareness (‘I had no awareness of services that were available in Australia’) and systematic barriers (‘long wait time and limited availability of certain healthcare service in remote areas’) limiting accessibility of SRH care. Further, most mentioned preferences for confidential services with private waiting areas, hiding their identity from others who may also be seeking medical care.

Provider preferences were clear: a nearly universal preference for providers of the same gender. Views on cultural background were mixed, some wanting providers from outside their community to preserve confidentiality, others wanting cultural similarity for understanding.

### 3.9. Migration’s Complex Impact

Women described how initial settlement stress negatively impacted sexual function: ‘When we were new here, we were under a lot of stress, like, looking for a place and work. We weren’t probably doing much.’ However, once settled, many described increased sexual autonomy. One reflected: ‘Now I live alone in a first-world country, and I can make my own decisions. There’s no family asking where I’m going, no [social] police to be afraid of. But it’s interesting because even with all this freedom, those traditional beliefs are still deep inside me.’

Similarly for men, migration was an incredibly stressful and difficult experience, placing SRH care at the bottom of a long list of priorities (‘I was very stressed at the time’). Most men were confused with the Australian healthcare system and lacked confidence in posing questions to professionals, not knowing where to go for SRH-specific care (‘I wasn’t sure if I could just approach doctor with very personal issues’). Overtime, confidence and understanding grew amongst the men, with many reflecting on a difficult but rewarding journey in achieving health confidence (‘building the ongoing relationship has made a big difference to my confidence and health’).

## 4. Discussion

These two qualitative studies taught us some important lessons for future qualitative research and healthcare practices addressing the sexual health needs of men and women from migrant backgrounds. Table 1 provides an overview of the lessons learnt through this research and the ways that research and healthcare providers should respond to these lessons.

### 4.1. The Complex Dynamics of Language Preference in Sexual Health Disclosure

We importantly found that language functions as more than a communication tool in sensitive health contexts for both men and women. In both qualitative projects, participants chose English despite available interpreters in their languages, yet simultaneously identified language as a healthcare barrier. This has implications for healthcare delivery beyond simply providing interpreters. Similarly to our qualitative interview findings, further research shows that even when professional interpreters are available, individuals from refugee backgrounds may decline their use. This choice is commonly due to confidentiality concerns, fear of judgement from interpreters sharing their cultural background, and discomfort discussing sexual health through a third party from their community [22]. The preference for English among participants in both studies may reflect what Pavlenko [23] describes as the emotional distance afforded by a second language. For our participants, this distance appeared to function not as a barrier but as a protective space—one that made discussing sexual health feel possible in English when it felt impossible in their mother tongue. This suggests that language choice in research and clinical settings is not simply a matter of comprehension, but a complex negotiation between cultural identity, emotional safety, and disclosure comfort. Providers and researchers should therefore not assume that offering native language support will be preferred, but rather create space for participants and patients to choose the language in which they feel most able to speak.

Providers must recognise that language choice reflects complex negotiations between cultural identity and disclosure comfort and respond to all individuals with sensitivity and understanding. Future research involving qualitative collection of migrants and refugees’ experiences and opinions must also ensure to continually provide opportunities for different language groups, explicitly outlining to each potential participant that translators can be present if preferred.

Enabling additional time for migrants and refugees to express themselves freely in whichever language they choose is equally important for migrant and refugee populations. Therefore, providers and researchers should consider booking in for longer consultation or interview time to account for any challenges. Many participants took additional time to ‘find the English word’ for SRH concepts and concerns, which required patience and kindness from the interviewer. Healthcare workers and future research must ensure they understand that, even when choosing to speak English, some may struggle to accurately articulate themselves in the way in which they see best and offer patience, understanding and kindness to their client.

### 4.2. Distinguishing Trauma-Related Silence from Knowledge Gaps in Sexual Health

Beyond language, the difficulty the participants experienced discussing certain topics reveals different barriers requiring different responses. Particularly for women, FGM’s unspeakability reflects deep cultural sensitivity and often unprocessed trauma. Research shows that 87.5% of women with FGM experienced hurtful comments from health professionals, whilst many encountered non-verbal expressions of surprise (78%) and disgust (55.1%), reactions that caused shame and acted as barriers to future care-seeking [24,25]. This requires trauma-informed approaches that respect silence whilst creating space for disclosure. Trauma-informed training should thus be central to healthcare professional training and ongoing professional development. In contrast, confusion about orgasm for women reflects knowledge gaps and an absence of intergenerational knowledge transfer. Research and healthcare systems must address both trauma-related silence and knowledge gaps, understanding that each individual will have different views, experiences and understandings of SRH and, thus, a single-intervention approach may not effectively function in these settings. In this study’s qualitative interviews with men, they seemed unsure of where to start when initially addressing SRH information. However, men were quickly able to actively engage in such conversations once initially prompted by the interviewer. It is important to recognise that discussing sensitive topics, including SRH, with any individual must be initiated with sensitivity, ensuring that a trusting relationship has been pre-established where the particpant or patient feels trusted, protected and equal to the researcher or healthcare provider. Building trusting relationships with migrant and refugee patients and research particpants may take additional time as compared to non-migrants. Repeated consultations and qualitative interviews with the same provider or researcher may be needed to gradually build up to discussions of SRH. Providing migrants and refugees with adequate, and easily digestible, information regarding confidentiality of sensitive information will assist in building trust in disclosing sensitive and personal information.

### 4.3. Gendered Power Dynamics and Sexual Communication in Intimate Relationships

Moreover, we found that women encountered difficulties in SRH communication within intimate relationships. Women’s difficulty communicating sexual needs with partners reveals how gender power dynamics shape intimate experiences. Rather than articulating discomfort, women described pulling away physically and hoping their partners would eventually learn what hurt them. This is not just about individual communication problems; it reflects how women are socialised to prioritise men’s feelings over their own physical comfort and needs. Migration creates new freedoms for many women, but our findings show that internalised beliefs about gender roles persist even after moving to a new country. Hawkey et al. (2018) similarly found that patriarchal control over women’s bodies continues after migration, with some women resorting to secret contraceptive use because direct communication with husbands remained impossible [26]. Women in the current study who described feeling more autonomous after migration still struggled to voice sexual dissatisfaction, worried that discussing issues like a lack of orgasm would hurt their partner’s feelings. This positions women as emotional caretakers even when experiencing their own distress. Education of both men and women regarding sexual pleasure and communication could assist in empowering open and active communication between intimate partners. In contrast, male participants did not largely acknowledge the role of female pleasure during sexual relationships, rather focusing on their own SRH and wellbeing during interviews. Some men mentioned good sexual health in terms of both them and their partner being able to carry children, reflecting on the importance of women for reproductive purposes. However, the focus of the male interviews was not on sexual satisfaction or partnerships, which may explain why these conversations were not naturally occurring or discussed in depth.

Whilst it is vital to not stereotype any individual based on their background, future researchers and healthcare providers must remain aware of these gender norms and the interaction they have with an individual’s health and wellbeing. As seen in our qualitative interviews, many women felt unheard by their male sexual partners and thus may have a very different relationship with sexual communication and openness as others. It is important to remain patient and kind during all sensitive conversations, ensuring calm environments are maintained. Furthermore, it is important for researchers and healthcare providers to remain non-judgemental and continually aware that silence does not always equate to absense of need, but rather could reflect cultural gender norms that shape communication. Therefore, patient-led communication cannot always be relied upon. Instead, it is important for healthcare providers and researchers to explore sensitive SRH topics with normalising promts and indirect questions [27].

Additionally, couple-based sexual health programmes must address the gendered power structures that silence men and women, not just provide tools for ‘better communication’. Healthcare interventions need to recognise the power dynamics that exists in many heterosexual relationships rather than assume that communication skills training alone will enable men and women to express their sexual needs and concerns.

### 4.4. The Role of Provider Gender in Facilitating Sexual Health Disclosure

Supporting the findings from both qualitative projects discussed here, previous research has demonstrated that migrant men and women primarily prefer to seek healthcare, particularly SRH, from providers of the same gender [5,28,29]. These preferences are commonly attributed to feelings of comfort and safety, as well as the perceived importance of biological understanding and the need to hide taboo topics from the opposite gender [18,29]. Cultural norms around privacy and gender roles may make interactions with male providers feel inappropriate, especially when they are unfamiliar or from a different cultural background [30]. Literature has also shown how common migration experiences of gender discrimination, racism or gender-based abuse heighten feelings of vulnerability around male professionals [31].

It is important for future researchers and healthcare providers to acknowledge the gender priorities of many migrant and refugee men and women and ensure that male and female workers are available to the client. Furthermore, this must be extended to translators and any other personnel that may be involved in their care or experience. Whilst none of the men in our qualitative study requested a male interviewer, it is important to acknowledge that some may prefer to disclose sensitive information to men only. Additionally, it cannot be known if the men may have disclosed further information to a man instead of the female interviewer. Offering the patient the option of either gender is important for building trust and comfort during sensitive conversations and should be implemented in all research and healthcare settings involving individuals from migrant and refugee backgrounds.

### 4.5. The Intersectionality of Individual, Relational and Structural Barriers to Sexual Healthcare

**Figure 1:**
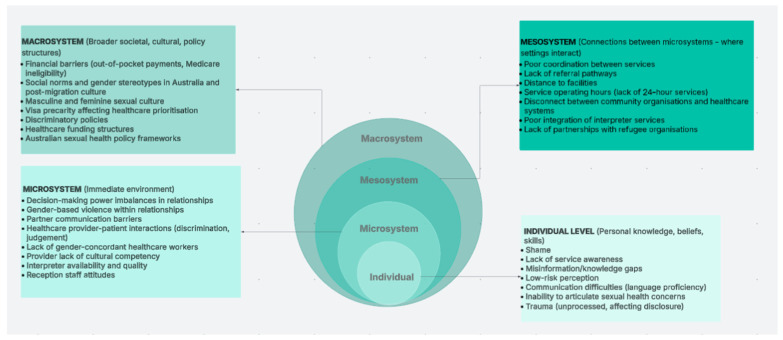
Intersectional relationship between individual, social and structural barriers in migrants and refugees accessing SRH care.

These individual and relational challenges exist within broader structural contexts that fundamentally shape access to care. The healthcare barriers migrant and refugee men and women face reflect structural inequities operating across multiple levels of the socio-ecological model (Figure 1). Prior research on refugee and migrant healthcare experiences underscores how barriers to care operate across multiple, intersecting levels, including individual, social, community and institutional contexts, with each playing a critical role in shaping SRH care engagement and outcomes, as seen in Figure 1 [5,32,33,34].

Our findings exemplify this intersection, for example: at the individual level, men and women similarly described difficulties articulating SRH concerns due to a multitude of individual and culturally informed barriers. These challenges extend into the microsystem, where access to culturally competent and gender-concordant providers is not always possible or prioritised by services, deterring engagement with SRH services. This intersects with the mesosystem, where limited collaboration between SRH services and community organisations—who assist migrants to find and access appropriate services—further constrains pathways to appropriate care opportunities. Finally, the macrosystem of broader structures continues to shape inequitable SRH access, with gaps such as the absence of mandated cultural competency training for Australian SRH professionals contributing to ongoing disconnects between providers and migrant communities. This can heighten anxiety and inhibit individuals’ ability to confidently communicate their SRH needs and concerns in professional settings. This interplay ultimately reinforces existing inequalities, contributing to experiences of discrimination and the perpetuation of cultural stereotypes within SRH encounters.

Addressing SRH inequities therefore requires person-centred care, training on cultural competency and compassion for healthcare personnel, involvement of family members and communities in care when applicable, and crucially, increasing financial access for migrant and refugee healthcare. These require structural and institutional changes to policy and practice that implement greater levels of financial support for all those who have migrated to Australia through refugee-like experiences. All healthcare workers, including those in people-facing roles such as receptionists, should undertake cultural competency training as part of their induction processes. Leveraging from community partnerships with organisations such as STTARS can be useful in managing and maintaining meaningful trainings.

Moreover, healthcare must support migrants’ own negotiations of what sexual wellbeing means within their particular intersections of culture, religion, relationship context, and personal values. Providing a calm, welcoming and confidential environment for all healthcare consultations is essential in ensuring that migrants and refugees feel comfortable and confident in conversations around their sexual wellbeing and personal views of SRH. Furthermore, it is also our responsibility to provide all migrants and refugees with the knowledge and understanding of Australian laws, especially around patient–provider confidentiality and cultural practices. Providing this information upon arrival to Australia, through culturally appropriate in-person conversations or written and illustrated handbooks, is essential in giving migrants and refugees the opportunity to explore a new culture whilst also having the confidence and freedom to practice their own beliefs [35]. This self-exploration is essential in supporting positive SRH development and confidence [35]. Engagement with migrant and refugee groups and partner organisations such as STTARS is essential in developing a framework for these tools.

### 4.6. The Important Role of Refugee-Specific Organisations in Research and Healthcare Engagement

The intensive STTARS partnership in the male qualitative studies demonstrates that community organisations hold methodological expertise researchers lack, particularly regarding culturally appropriate approaches to sensitive topics. STTARS’ knowledge that foregrounding sexual health would deter men, that casual atmospheres reduce anxiety, and that gradual integration works better than upfront disclosure reflects years of trust-building and cultural learning within communities. This challenges traditional research hierarchies where academic researchers design studies and communities simply provide access. Cyril et al. (2015) argue that genuine collaborative partnerships require community knowledge to shape fundamental design decisions rather than simply inform recruitment strategies [36]. Our experience supports this, though we note that such partnerships require resources and timeframes often incompatible with standard grant structures, suggesting that funding models must change to support authentic collaboration. In healthcare settings, partnerships with migrant- and refugee-specific organisations, such as STTARS, should be better-used as a resource of learning and engagement. These organisations can be used to provide education resources and workshops to healthcare staff, teaching others key concepts of trauma-informed care and culturally sensitive conversations. The strong relationship that members of STTARS hold with migrant and refugee men was helpful in recruiting participants for our qualitative work, with staff members promoting the work and researchers with their own clients. Similarly, in healthcare settings, partnerships with STTARS can help uncertain migrants and refugees to trust in healthcare practitioners that are referred to by STTARS staff. This, in turn, can assist in increasing consistent and meaningful engagement with healthcare services.

### 4.7. Study Limitations

We must acknowledge a critical limitation with profound implications for research equity. The populations excluded from both studies are precisely those experiencing greatest sexual health inequities: recent arrivals with limited English, those without Medicare facing severe financial constraints, those most socially isolated within communities where sexual health remains taboo, and those with a precarious visa status avoiding any formal systems. This is not simply a study limitation but a research equity issue. This pattern is not unique to our work. Research has consistently shown that volunteer bias is particularly pronounced in sexuality research, with those who choose to participate typically reporting more openness to sexual topics and less shame than those who do not [37]. Similarly, achieving representative samples in refugee and asylum-seeker research remains a persistent methodological challenge, with the majority of studies in this field drawing on more accessible, and therefore less marginalised, subgroups of these communities [38]. Even well-resourced recruitment efforts targeting migrant populations in sexual health research have documented the difficulty of reaching those most affected by structural barriers [39]. Our findings should therefore be interpreted with this in mind: they most likely reflect the experiences of migrants who were already relatively comfortable engaging with SRH topics and formal research processes.

Our findings mean that evidence bases for sexual health policy are built primarily on experiences of more privileged migrants, risking interventions that do not address the needs of those facing the greatest barriers. Addressing this requires fundamental methodological innovation: community-led research, creative non-interview methods, intensive support that recognises participation as labour deserving substantial compensation, and willingness to work with communities in their own languages and contexts rather than expecting them to come to researchers [40]. Finally, it is important to acknowledge that the two interview studies employed slightly different methodological approaches. However, both were semi-structured in design and conducted by researchers with comparable qualitative research backgrounds and training, making it reasonable to assume a degree of consistency in interview style across the two studies. It must also be highlighted that this work is reflective in nature. Whilst the two qualitative studies were designed with replicability in mind, no two interviews are identical and no two researchers’ reflections of the same encounter will be precisely the same.

## 5. Conclusions

Our findings demonstrate that research challenges mirror healthcare challenges, suggesting that methodological insights translate directly to clinical practice. If participants struggled to articulate sexual experiences in research contexts designed to be supportive, clinical encounters with time constraints and power imbalances will face greater challenges. Healthcare improvements therefore require the same principles that enhance research: gradual approaches to sensitive topics, normalising discomfort, multiple communication modalities, explicit trust-building, and recognition that structural barriers require systemic solutions rather than an individual provider’s cultural competence [41]. Future research must move beyond describing challenges to testing interventions that address identified needs whilst centring community voices in defining what sexual health and wellbeing mean within their diverse contexts.

Several key insights emerged from comparing experiences. First, neither upfront nor delayed disclosure of inclusion of SRH topics is inherently superior for research recruitment. Second, offering multilingual support matters symbolically even when unutilised. Third, intensive community partnerships enable cultural expertise to shape fundamental design decisions. Fourth, flexible formats with anonymity options proved particularly valuable. Fifth, some topics remained extremely difficult regardless of rapport, and this difficulty itself represents important data. Finally, both components likely excluded the most marginalised: those with limited English, no Medicare, social isolation, or highest visa precarity, meaning findings represent more privileged subsets of migrant experiences.

## Figures and Tables

**Table 1:** Overview of the lessons learnt and how they apply to healthcare and research settings.

Lessons Learnt	Application to Healthcare and Research
The complex dynamics of language preference in sexual health disclosure	Ensure provision of trained interpreters Sensitive and patient response to language choices Longer consultation/interview time
Distinguishing trauma-related silence from knowledge gaps in sexual health	Ensure trauma-informed training is provided to all healthcare workers Understanding that single-intervention approaches are not universal Trusting relationships are important for SRH disclosure Adequate and easily digestible information around confidentiality needed
Gendered power dynamics and sexual communication in intimate relationships	SRH education to men and women based on the importance of SRH communication in intimate relationships Calming environments for interviews/consultations Normalising prompts and indirect questions to aid exploration of SRH topics
The role of provider gender in facilitating sexual health disclosure	Availability of gender-specific health providers and researchers Availability of translators of the same gender as clients
The intersectionality of individual, relational and structural barriers to sexual healthcare	Person-centred care Training on cultural competency for all healthcare providers and researchers in the field Involvment of families and communities in SRH care if applicable.
The important role of refugee-specific organisations in research and healthcare engagement	Collaboration with refugee-specific organisations to establish interview protocols and healthcare environments Organisations can be used to help support patient–provider relationships

## Data Availability

The data presented for the womens component of this study is avalaibale at: https://doi.org/10.1186/s12939-025-02614-z. Data presented for the mens component is curently not published but further inquiries can be directed to the corresponding author.

## References

[1] Australian Human Rights Commission (2025). Stats & Facts: Refugees and People Seeking Asylum. https://humanrights.gov.au/human-rights-education/stats-and-facts-about-discrimination/statistics-about-refugees-and-people-seeking-asylum.

[2] Castleton P., Chaudhry A.S., Damabi N., Meherali S., Lassi Z.S. (2025). Crossing Borders: SRH Challenges Among Immigrant and Minority Adolescents. Int. J. Environ. Res. Public Health.

[3] Australian Institute of Health and Welfare (2023). Health of Refugees and Humanitarian Entrants in Australia. https://www.aihw.gov.au/reports/cald-australians/health-of-refugees-and-humanitarian-entrants.

[4] Khatri R.B., Assefa Y. (2022). Access to health services among culturally and linguistically diverse populations in the Australian universal health care system: Issues and challenges. BMC Public Health.

[5] Castleton P., Mirzaei Damabi N., Begum M., Mengesha Z., Lassi Z.S. (2025). The influence of gender norms on post-migration men’s sexual and reproductive health: A scoping review. PLoS ONE.

[6] Lassi Z.S., Damabi N.M., Begum M., Avery J.C., Meherali S. (2025). Breaking the silence: Addressing sexual health challenges among migrant and refugee women. Women’s Health.

[7] Metusela C., Ussher J., Perz J., Hawkey A., Morrow M., Narchal R., Estoesta J., Monteiro M. (2017). “In My Culture, We Don’t Know Anything About That”: Sexual and Reproductive Health of Migrant and Refugee Women. Int. J. Behav. Med..

[8] Napier-Raman S., Bidewell J., Hossain S.Z., Mpofu E., Lee M.-J., Liamputtong P., Dune T. (2025). Migrant and Refugee Youth’s Sexual and Reproductive Health and Rights: A Gender Comparison of Knowledge, Behaviour, and Experiences. Sex. Cult..

[9] Botfield J.R., Zwi A.B., Rutherford A., Newman C.E. (2018). Learning about sex and relationships among migrant and refugee young people in Sydney, Australia: ‘I never got the talk about the birds and the bees’. Sex Educ..

[10] Ellawela Y., Nilaweera I., Holton S., Rowe H., Kirkman M., Jordan L., McNamee K., Bayly C., McBain J., Sinnott V. (2017). Contraceptive use and contraceptive health care needs among Sri Lankan migrants living in Australia: Findings from the understanding fertility management in contemporary Australia survey. Sex. Reprod. Healthc..

[11] Inthavong A.B., Pourmarzi D. (2024). Characteristics of Sexual Health Programs for Migrants, Refugees, and Asylum Seekers: A Scoping Review. Int. J. Environ. Res. Public Health.

[12] Agu J., Lobo R., Crawford G., Chigwada B. (2016). Migrant Sexual Health Help-Seeking and Experiences of Stigmatization and Discrimination in Perth, Western Australia: Exploring Barriers and Enablers. Int. J. Environ. Res. Public Health.

[13] Kennedy S., Kidd M.P., McDonald J.T., Biddle N. (2015). The Healthy Immigrant Effect: Patterns and Evidence from Four Countries. J. Int. Migr. Integr..

[14] Cayreyre L., Korchia T., Loundou A., Jego M., Théry D., Berbis J., Gentile G., Auquier P., Khouani J. (2024). Lifetime sexual violence experienced by women asylum seekers and refugees hosted in high-income countries: Literature review and meta-analysis. J. Forensic Legal Med..

[15] Mah H., Dobson R., Thomson A. (2025). The Importance of Lived Experience: A Scoping Review on the Value of Patient and Public Involvement in Health Research. Health Expect..

[16] Hodges E., Leditschke A., Solonsch L. (2023). The Lived Experience Governance Framework: Centring People, Identity and Human Rights for the Benefit of All; Prepared by LELAN (SA Lived Experience Leadership & Advocacy Network) for the National Mental Health Consumer and Carer Forum and the National PHN Mental Health Lived Experience Engagement Network.

[17] Davidson N., Hammarberg K., Fisher J. (2024). Ethical Considerations in Research with People from Refugee and Asylum Seeker Backgrounds: A Systematic Review of National and International Ethics Guidelines. J. Bioeth. Inq..

[18] Mirzaei Damabi N., Avery J.C., Begum M., Meherali S., Lassi Z.S. (2025). Redefining intimacy: A qualitative study on sexual function experiences and perspectives among migrant and refugee women in South Australia. Int. J. Equity Health.

[19] Akmalia F. (2024). Qualitative Literacy: A Guide to Evaluating Ethnographic and Interview Research. Qual. Rep..

[20] Braun V., Clarke V. (2019). Reflecting on reflexive thematic analysis. Qual. Res. Sport Exerc. Health.

[21] Olmos-Vega F.M., Stalmeijer R.E., Varpio L., Kahlke R. (2023). A practical guide to reflexivity in qualitative research: AMEE Guide No. 149. Med. Teach..

[22] Mengesha Z.B., Perz J., Dune T., Ussher J. (2018). Talking about sexual and reproductive health through interpreters: The experiences of health care professionals consulting refugee and migrant women. Sex. Reprod. Healthc..

[23] Pavlenko A. (2012). Multilingualism and emotions. The Routledge Handbook of Multilingualism.

[24] Chalmers B., Hashi K.O. (2000). 432 Somali women’s birth experiences in Canada after earlier female genital mutilation. Birth.

[25] Wood R., Richens Y., Lavender T. (2021). The experiences and psychological outcomes for pregnant women who have had FGM: A systematic review. Sex. Reprod. Healthc..

[26] Hawkey A.J., Ussher J.M., Perz J. (2018). “If you don’t have a baby, you can’t be in our culture”: Migrant and refugee women’s experiences and constructions of fertility and fertility control. Women’s Reprod. Health.

[27] Sharman R., Allen A., van Niekerk K., Coles A., Manocha R., Foran T. (2024). “What Is Normal?”: A Qualitative Exploration of Health Practitioners’ Reports of Treating Patients Presenting with Unpleasant Sexual Experiences. Arch. Sex. Behav..

[28] Aubrey C., Mumtaz Z., Patterson P.B., Chari R., Mitchell B.F.P. (2017). Accommodating Immigrant Women’s Preferences for Female Health Care Providers. Obstet. Gynecol..

[29] Hawkey A.J., Ussher J.M., Perz J. (2022). What do women want? Migrant and refugee women’s preferences for the delivery of sexual and reproductive healthcare and information. Ethnicity Health.

[30] Ouahid H., Sebbani M., Cherkaoui M., Amine M., Adarmouch L. (2025). The influence of gender norms on women’s sexual and reproductive health outcomes: A systematic review. BMC Women’s Health.

[31] Guruge S., Sidani S., Illesinghe V., Younes R., Bukhari H., Altenberg J., Rashid M., Fredericks S. (2018). Healthcare needs and health service utilization by Syrian refugee women in Toronto. Confl. Health.

[32] Darebo T.D., Spigt M., Teklewold B., Badacho A.S., Mayer N., Teklewold M. (2024). The sexual and reproductive healthcare challenges when dealing with female migrants and refugees in low and middle-income countries (a qualitative evidence synthesis). BMC Public Health.

[33] Taylor J., Lamaro Haintz G. (2018). Influence of the social determinants of health on access to healthcare services among refugees in Australia. Aust. J. Prim. Health.

[34] WHO (2022). Refugee and Migrant Health. https://www.who.int/news-room/fact-sheets/detail/refugee-and-migrant-health?utm_source.

[35] Stirling-Cameron E., Almukhaini S., Dol J., DuPlessis B.J., Stone K., Aston M., Goldenberg S.M. (2024). Access and use of sexual and reproductive health services among asylum-seeking and refugee women in high-income countries: A scoping review. PLoS ONE.

[36] Cyril S., Smith B.J., Possamai-Inesedy A., Renzaho A.M.N. (2015). Exploring the role of community engagement in improving the health of disadvantaged populations: A systematic review. Glob. Health Action.

[37] Strassberg D.S., Lowe K. (1995). Volunteer bias in sexuality research. Arch. Sex. Behav..

[38] Enticott J.C., Shawyer F., Vasi S., Buck K., Cheng I.H., Russell G., Kakuma R., Minas H., Meadows G. (2017). A systematic review of studies with a representative sample of refugees and asylum seekers living in the community for participation in mental health research. BMC Med. Res. Methodol..

[39] Vujcich D., Brown G., Durham J., Gu Z., Hartley L., Lobo R., Mao L., Moro P., Pillay V., Mullens A.B. (2022). Strategies for Recruiting Migrants to Participate in a Sexual Health Survey: Methods, Results, and Lessons. Int. J. Environ. Res. Public Health.

[40] Liamputtong P. (2023). How to Conduct Qualitative Research in Social Science.

[41] Mengesha Z.B., Dune T., Perz J. (2016). Culturally and linguistically diverse women’s views and experiences of accessing sexual and reproductive health care in Australia: A systematic review. Sex. Health.

